# Blue and Long-Wave Ultraviolet Light Induce *in vitro* Neutrophil Extracellular Trap (NET) Formation

**DOI:** 10.3389/fimmu.2019.02428

**Published:** 2019-10-25

**Authors:** Elsa Neubert, Katharina Marie Bach, Julia Busse, Ivan Bogeski, Michael P. Schön, Sebastian Kruss, Luise Erpenbeck

**Affiliations:** ^1^Department of Dermatology, Venereology and Allergology, University Medical Center Göttingen, Göttingen, Germany; ^2^Institute of Physical Chemistry, Göttingen University, Göttingen, Germany; ^3^Institute of Cardiovascular Physiology, University Medical Center Göttingen, Göttingen, Germany; ^4^Lower Saxony Institute of Occupational Dermatology, University Medical Center Göttingen, Göttingen, Germany; ^5^Center for Nanoscale Microscopy and Molecular Physiology of the Brain (CNMPB), Göttingen, Germany

**Keywords:** neutrophilic granulocytes, NET formation, UV-Vis light, inflammation, ROS formation, riboflavin

## Abstract

Neutrophil Extracellular Traps (NETs) are produced by neutrophilic granulocytes and consist of decondensed chromatin decorated with antimicrobial peptides. They defend the organism against intruders and are released upon various stimuli including pathogens, mediators of inflammation, or chemical triggers. NET formation is also involved in inflammatory, cardiovascular, malignant diseases, and autoimmune disorders like rheumatoid arthritis, psoriasis, or systemic lupus erythematosus (SLE). In many autoimmune diseases like SLE or dermatomyositis, light of the ultraviolet-visible (UV-VIS) spectrum is well-known to trigger and aggravate disease severity. However, the underlying connection between NET formation, light exposure, and disease exacerbation remains elusive. We studied the effect of UVA (375 nm), blue (470 nm) and green (565 nm) light on NETosis in human neutrophils *ex vivo*. Our results show a dose- and wavelength-dependent induction of NETosis. Light-induced NETosis depended on the generation of extracellular reactive oxygen species (ROS) induced by riboflavin excitation and its subsequent reaction with tryptophan. The light-induced NETosis required both neutrophil elastase (NE) as well as myeloperoxidase (MPO) activation and induced histone citrullination. These findings suggest that NET formation as a response to light could be the hitherto missing link between elevated susceptibility to NET formation in autoimmune patients and photosensitivity for example in SLE and dermatomyositis patients. This novel connection could provide a clue for a deeper understanding of light-sensitive diseases in general and for the development of new pharmacological strategies to avoid disease exacerbation upon light exposure.

## Introduction

Neutrophilic granulocytes (hereafter referred to as neutrophils) are able to expel fibril networks of decondensed chromatin, decorated with a variety of antimicrobial substances, in a process termed neutrophil extracellular trap (NET) formation or NETosis (1). Initially, NETosis was described as an immune defense strategy against intruding pathogens, distinct from phagocytosis and the release of cytotoxic substances. Apart from their role in the defense within the innate immune system, the dysregulation of NETosis appears to be involved in the pathology of various diseases (2) such as rheumatoid arthritis (3), systemic lupus erythematosus (SLE) (4), psoriasis (5, 6), thrombosis (7), atherosclerosis (8), and cancer (9). The activation mechanisms and underlying cascades of NETosis depend highly on the particular stimulus (10, 11). Additionally, neutrophils and therefore also neutrophils undergoing NETosis, are very sensitive to environmental cues that affect, for example, adhesion (12–15).

In most scenarios, the cell undergoes a characteristic sequence of morphological changes during NETosis including chromatin decondensation, cytoskeleton degradation, cell rounding, and softening, which ultimately lead to NET expulsion and cell death (“suicidal” NETosis) (16, 17). Initially, active enzyme-dependent mechanisms dominate these processes. For instance, the initiation of chromatin decondensation often involves the release of neutrophil elastase (NE) and myeloperoxidase (MPO) from the neutrophilic granules and subsequent translocation to the nucleus (18, 19). Following initiation of chromatin decondensation, which represents the point of no return in NETosis, further progression until the NET release is mainly driven by the material properties of the NETotic cell such as the entropic swelling of its chromatin (17).

Interestingly, a connection between dysregulated NET formation and the production of autoantibodies against NET components has been described in several diseases including SLE, rheumatoid arthritis and small-vessel vasculitis (20, 21). Mechanistically, NET formation in this context often relies on the activity of peptidylarginine deiminase 4 (PAD4), which citrullinates histones contributing to chromatin decondensation (22–25). This hypercitrullination has been linked to the development of autoantigens against citrullinated histones, for instance in the pathogenesis of rheumatoid arthritis (26). Interestingly, autoimmune disorders such as systemic lupus erythematosus (SLE) or dermatomyositis, can also be triggered and/or aggravated by light. Although for these diseases both the increased propensity for NET formation as well as the marked light sensitivity is well-documented (27–30), the connection between these two phenomena remains elusive.

Electromagnetic radiation of wavelengths above ultraviolet C (UVC) light passes the ozone layer of the stratosphere and can thus reach the human skin (31). Within the human skin, light intensity is modified by reflection, absorption as well as scattering and its penetration depth increases with higher wavelengths (32–34). However, the actual penetration of each wavelength also strongly depends on the specific skin composition, as well as body region, age, gender, skin type, pigmentation, and therefore ethnicity.

High-energy UV light causes severe skin damage. This has been linked not only to photodermatoses but also to phototoxic and photoallergic reactions, skin cancer and photoaging (35, 36). Many of these reactions are mediated by highly reactive radicals and/or reactive oxygen species (ROS) originating from the excitation of photosensitive substances (37). Prominent examples are flavin-based molecules originating from riboflavin (also known as vitamin B2) (38). Under physiological conditions, these reactions are kept in balance by antioxidants, but they can be strongly dysregulated in the context of diseases and after persistent exposure to UV light.

Additionally, light can have several direct effects on neutrophils. Irradiation with UVB light has been reported to recruit neutrophils into upper layers of the skin and has been linked to photoaging (35, 39). Furthermore, increased apoptosis rates of neutrophils occur upon direct irradiation with high doses of UVB or UVC (40, 41) and UVC light can also induce a unique form of NADPH oxidase (NOX)-independent NETosis (named apoNETosis) (41). However, the connection between NET formation and light in a physiologically relevant setting with light which penetrates deeper into the skin such as UVA or blue light, has not been investigated. Thus, a deeper understanding of direct effects of light on immune cells could greatly add to our understanding of light-induced or -aggravated diseases and facilitate the development of therapeutic strategies.

## Materials and Methods

### Isolation of Neutrophils

All experiments with human neutrophils were approved by the Ethics Committee of the University Medical Center (UMG) Göttingen (protocol number: 29/1/17). Neutrophils were isolated from fresh venous blood of healthy donors. Beforehand, all donors were fully informed about possible risks, and their informed consent was obtained in writing. The consent could be withdrawn at any time during the study. Blood was collected in S-Monovettes EDTA (7.5 ml, Sarstedt), and neutrophils were isolated according to previously published protocols based on histopaque 1119 (Sigma Aldrich) as well as Percoll (GE Healthcare) density gradients (17, 42). Neutrophils were resuspended in HBSS^−Ca2+/Mg2+^ and further diluted in the desired medium as described in the appropriate methods sections and figure legends. Purity of the cell preparation was >95% as assessed by cytospin (Cytospin 2 centrifuge, Shanson) and Diff Quick staining (Medion, Diagnostics).

### Irradiation of Neutrophils With LED Light

Neutrophils were suspended in either Roswell Park Memorial Institute (RPMI) comp. [RPMI without phenol red (Gibco) + 0.5% heat-inactivated (at 56°C) fetal calf serum (hiFCS, Biochrom GmbH, Merck Millipore)] ± 10 mM HEPES (Roth) or Hank's balanced salt solution (HBSS) comp. [HBSS^+Ca2+/Mg2+^ without phenol red (Lonza) containing 0.5% hiFCS and glucose (AppliChem) equalized to RPMI]. If applicable, these media were supplemented with 0.2 or 2 mg/l riboflavin (Sigma-Aldrich) and 1 mM tryptophan (Sigma-Aldrich) as indicated in the figure captions. Cells were seeded at 10,000 cells per well in CELLview^TH^ black glass-10-well-slides (Greiner bio-one) and left to settle for 30 min (37°C, 5% CO_2_). Afterward, the appropriate medium was added, and cells were irradiated with the indicated LED-light at 37°C (ibidi heating system). Cells were irradiated in the heating chamber from below with LEDs of 375 nm (ultraviolet light, M375L3 Mounted LED, Thorlabs GmbH), 470 nm (blue light, M470L3 Mounted LED, Thorlabs GmbH), or 565 nm (green light, M565L3 Mounted LED, Thorlabs GmbH), which were attached to an uncoated convex lens (PLANO-CONVEX LA1131, f = 50.0 mm, uncoated, Thorlabs GmbH) and a T-cube LED Driver (Thorlabs GmbH). For evaluation of light dose-dependent effects, cells were irradiated with cumulative doses of 3.5, 18, 35, and 70 J/cm^2^ at 375 nm or 21, 54, 107, and 214 J/cm^2^ at 470 nm. The light doses were calculated with respect to the actual power of the LED as measured with the PM12-122 Compact USB Power Meter (Thorlabs GmbH), taking into account the actual distance between the light source and the cells as well as the light transmission through the CELLview^TH^ glass-10-well-slides according to the manufacturer's specifications. For experiments with an equal light energy-dose or photon flux, the light-doses or duration of exposure, respectively, were adjusted for 470 and 565 nm, the reference value was irradiation with 70 J/cm^2^ at 375 nm. Exclusive treatment with the indicated medium without irradiation was used as a negative control and activation with 100 nM PMA (Sigma Aldrich) as a positive control. Before, during, and after activation with light or PMA, the cells were carefully shielded from other light sources. After the activation, the cells were incubated for 3 h, and NETosis was stopped by fixing the cells in 2% paraformaldehyde (PFA, Roth). Before further staining, the cells were kept at 4°C.

### Inhibitor Experiments

For inhibition experiments, cells were isolated, settled in RPMIcomp. supplemented with 10 mM HEPES and activated as described above. Inhibitors or ROS scavengers were added at least 20 min (in case of MitoTEMPO 1 h) before cell irradiation with 70 J/cm^2^ of 375 nm or 214 J/cm^2^ of 470 nm, at 37°C. For an additional control experiment, Trolox and catalase/SOD were added separately after irradiation. The cells were then incubated for an additional 3 h without an additional washing step in the presence of the inhibitors to allow for NET formation and fixed by 2% PFA. Pure medium or 100 nM PMA without irradiation were used as negative and positive controls, respectively. The following inhibitors and ROS scavengers were used in this study: GW-311616A hydrochloride (iNE, Axon Medchem) at 5 μM, 4-aminobenzoic acid hydrazide (4-ABAH, Cayman chemicals) at 100 μM, z-VAD-FMK (Promega) at 20 μM, necrostatin-1 (Nec-1, Enzo) at 50 μM, Y-27632-dihydrochloride (Abcam) at 20 μM, Cl-amidine (Merck Millipore) at 200 μM, MitoTEMPO (Sigma-Aldrich) at 5 μM, diphenyleneiodonium chloride (DPI, Sigma-Aldrich) at 1 μM, Trolox (Sigma-Aldrich) at 50 μM, PEG-catalase at 2,000 U/ml (Sigma-Aldrich), and a mixture of catalase (filtered, Worthington) and superoxide dismutase (SOD, Sigma Aldrich/Merck) at 2,000 and 50 U/ml, respectively.

### NET Quantification

To investigate levels of NETosis, cells were washed twice with PBS (Sigma-Aldrich) and, subsequently, neutrophilic DNA was stained with 1.62 μM Hoechst 33342 (Thermo Fisher Scientific) for 15 min. After staining, the cells were stored in PBS for further analysis. For blinded quantification, six microscopic fluorescence images (16 ×) were obtained in a standardized manner (Axiovert 200 equipped with EC Plan-Neofluar Ph1 and DAPI filter Set 49, Zeiss, software: Metamorph 6.3r2., Molecular Devices or Micro Manager 1.4) using the camera CoolSNAP ES (Photometrics). The number of decondensed vs. condensed nuclei was counted in these images using ImageJ 1.46r (National Institutes of Health), and the relative number of decondensed nuclei/expelled NETs was determined as a percentage of total cells (‘NETotic cells’) according to previously published studies (17, 43). Relative rates of NETotic cells were normalized to NETs after light-irradiation without any inhibitor (“Rel. number NETotic cells”). The amount of released NETs was determined by counting SYTOX Green-positive cells with decondensed nuclei.

### Live Cell Imaging/Discontinuous Irradiation of Neutrophils

**Figure 1:**
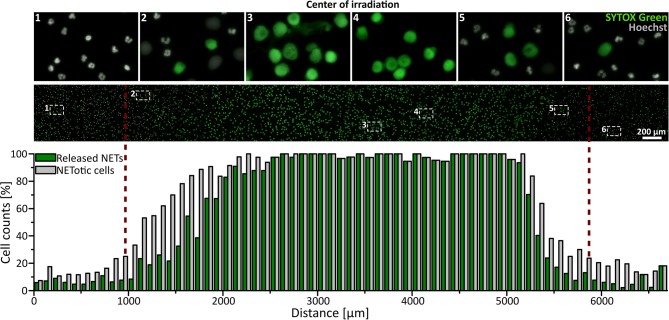
UVA light-induced, locally restricted decondensation of chromatin. UVA light (300–400 nm, ~60 J/cm^2^) leads to chromatin decondensation and expulsion of the chromatin into the extracellular space as indicated by SYTOX green positive staining of decondensed chromatin. This effect is locally restricted to the area of irradiation. Irradiation: 3 min to induce NET formation + intermittent irradiation during live-cell imaging (3 h; frame rate: 15 min/image). Cells were kept in RPMIcomp. + 10 mM HEPES.

Neutrophils (5 × 10^6^ per ml in RPMI + 0.5% FCS or HSA + 10 mM HEPES) were seeded in ibidi channel slides (μ-Slide l^0.6^ Luer, ibidi) and stained with 1.62 μM Hoechst and, if indicated, 5 μM SYTOX Green (life technologies) at 37°C for 10 min. Cells were irradiated with broad-spectrum UVA light (300–400 nm) for 3 min using the DAPI filter Set 49. For life cell imaging, NET formation was observed in real time for 3.5 h with a frame rate of one picture per min (Uniblitz stutter driver, model VCM-D1, Visitron Systems) and a 15 ms exposure time. To exclude the toxic effects of the photo-activation of Hoechst, a control experiment was performed without DNA staining during live cell imaging. In this case, NET rates were determined by Hoechst staining directly after 3.5 h. For The SYTOX Green/Hoechst double staining, NETosis was observed for 3 h with a frame-rate of 15 min per image. Images were recorded at 10 × or 16 × magnification. Images in the center of the light beam and in non-irradiated areas were obtained in a standardized pattern and in a blinded manner, and NETosis rates were determined as described above. The representative combined panorama image in Figure 1 was obtained with the Plugin MosaicJ for ImageJ (44).

### Immunofluorescence Staining

To confirm colocalization of MPO with decondensed chromatin and histone citrullination as a marker for NETosis, activated cells were analyzed by immunofluorescence according to previously published protocols (17). Here, a permeabilization buffer containing 0.1% tritonX and a BSA-based blocking solution (from TSA-kit, Perkin Elmer) was used. Cells were stained with the primary monoclonal anti-human antibody against myeloperoxidase (IgG1, mouse, clone:2C7, ab25989, 1:500, Abcam) and the polyclonal antibody against citrullinated histone 3 (H3Cit, rabbit, ab5103, 1:500, Abcam) and visualized with the polyclonal anti-mouse Alexa488 secondary antibody (IgG, goat, 1:300, #4408, Cell Signaling Technology) or the anti-rabbit Alexa555 secondary antibody (IgG, goat, 1:500, A211428, Life technologies), respectively. Directly before mounting with fluorescence mounting medium (Dako), DNA was stained with Hoechst. Colocalization of MPO, H3Cit and DNA was imaged at 100 × magnification by confocal fluorescence microscopy (IX83, Olympus; software: Olympus Fluoview Ver.4.2, Olympus). All pictures were recorded at equal exposure times for MPO and H3Cit within the same experiment, to ensure comparability.

### ROS (H_2_O_2_) Detection/AmplexRed Assay

Cells were seeded at 10,000 cells per well in RPMIcomp. + 10 mM HEPES, HBSScomp. or HBSScomp. + 2 mg/l riboflavin + 1 mM tryptophan and activated at 70 J/cm^2^ of 375 nm light. After activation, 5 μl samples of the supernatant were taken at defined time points (0, 10, 20, or 30 min) close to the slide bottom for reactive oxygen species (ROS) detection. As controls, cells in all three media were either left without irradiation, only media was irradiated, or cells were treated with 100 nM PMA without irradiation. The obtained samples were diluted in a black 96-well-plate (BRANDplates, BRAND GMBH) with PBS containing 50 μM of AmplexRed reagent (Thermo Fisher Scientific), a highly sensitive probe for H_2_O_2_, and 0.5 U/ml horseradish peroxidase (HRP, Sigma-Aldrich/Merck). Additionally, 10 U/ml SOD (Sigma Aldrich/Merck) were added, to ensure complete detection of ROS by transformation of superoxide radicals to H_2_O_2_. During the sample collection, cells were gently rocked to ensure equal distribution of ROS. For all samples, the fluorescence intensities of the formed resorufin were measured with the microplate reader Clario Star (software 5.40.R3, BMG labtech), and the results were processed with the software MARS (version 3.32, BMG labtech). Absolute H_2_O_2_ concentrations were determined *via* calibration with H_2_O_2_ (Roth) in HBSScomp. After ROS detection, cells were further incubated for a total of 3 h before terminating the activity with 2% PFA, and the number of NETotic cells was determined.

### Light Absorption by Riboflavin

The absorbance spectrum of riboflavin (Sigma-Aldrich) was obtained in PBS against PBS alone with the UV-VIS-NIR spectrometer (JASCO V-670, Spectra Manager Software) using a 10 mm-path cuvette.

### Statistics

Statistical analysis was performed using GraphPad Prism (version 6.0 for Mac or Windows, GraphPad Software Inc.). If applicable, GAUSS distribution was confirmed by the Shapiro-Wilk normality test. Significance was confirmed on unnormalized data by a two-tailed paired *t*-test or a one-way ANOVA/Bonferroni's multiple comparisons test with ^*^p < 0.05, ^**^p < 0.01, ^***^p < 0.001, ^****^p < 0.0001. Error = mean ± standard error of the mean (SEM) or standard deviation (SD), as indicated.

## Results

### UVA and Blue Light Induce NETosis Dose-Dependently

To investigate whether UVA light is sufficient to activate NETosis, freshly isolated human neutrophils were irradiated for 3 min with physiologically relevant broad-spectrum UVA light in a standard microscopy setup (wavelengths 300–400 nm, ~60 J/cm^2^). Morphological changes of the nuclei were recorded using Hoechst staining over 3.5 h in real-time (Supplementary Movie). Neutrophilic chromatin readily decondensed over time, rounded up and finally formed cloud-like structures of decondensed chromatin 1–2 h after exposure to light. This characteristic rearrangement of chromatin is consistent with previously published live-cell studies of NETosis (17, 45–47). The fully decondensed chromatin stained positive for SYTOX Green within 3 h, indicating cell membrane rupture and NET release. The counting of decondensed nuclei led to slightly higher cell counts than SYTOX Green-positive cells since not all NETotic cells had already released the final NET into the medium. This was particularly prominent in the transition zone between irradiated and non-irradiated regions. Strikingly, this dramatic effect was restricted to the light-exposed area and did not occur in unexposed areas (Figure 1) and was reproducible with neutrophils from different donors (Supplementary Figure 1). To exclude light-induced cytotoxic effects of the Hoechst staining, neutrophils were stained after the full incubation period as control (Supplementary Figure 1).

**Figure 2:**
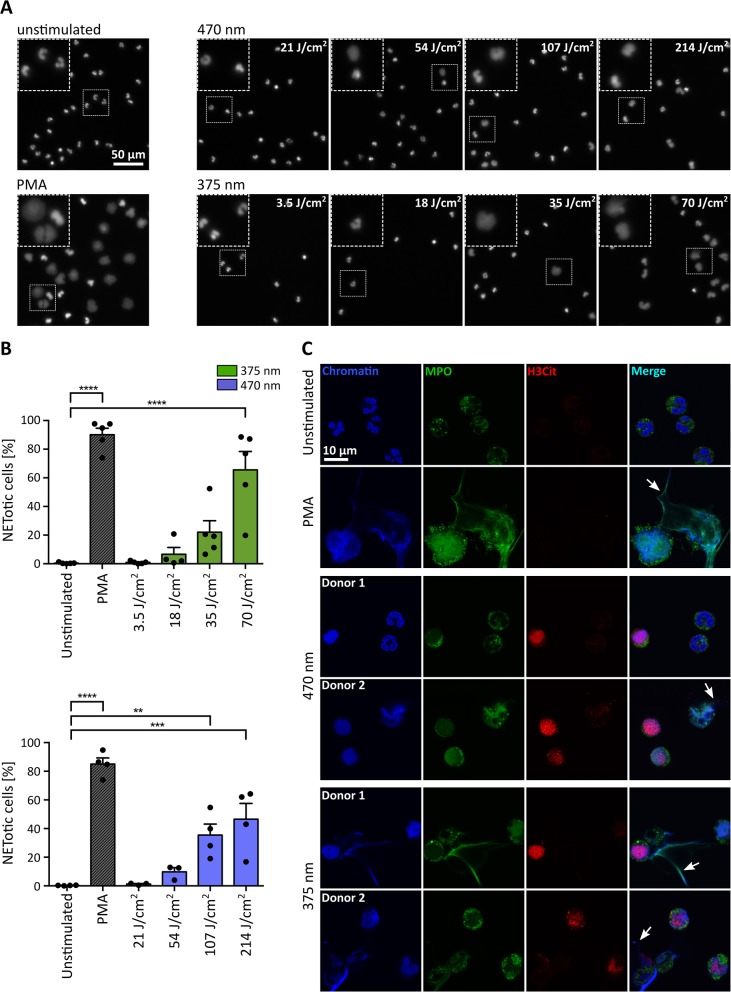
UVA and blue light induce the formation of NETs in a dose-dependent manner. **(A)** Representative fluorescence images of neutrophils exposed to different doses of LED-light [375 nm (3.5, 18, 35, and 70 J/cm^2^) or 470 nm (21, 54, 107, and 214 J/cm^2^)]. Decondensation of chromatin, stained by Hoechst, clearly increases with duration of light exposure. **(B)** NET rates significantly increase for both tested LEDs with light doses. Statistics: one-way-ANOVA with Bonferroni's multiple comparisons test (tested against unstimulated cells). ^**^p < 0.01, ^***^p < 0.001, ^****^p < 0.0001. *N* = 3–5 independent experiments. Error bars = SEM. **(C)** Histone 3 becomes citrullinated (red/Alexa555) in early stages of chromatin decondensation (blue/Hoechst) after irradiation with both wavelengths. The decondensed chromatin colocalizes with MPO (green/alexa488) most prominently within the released NET fibers (arrows), similar to PMA-induced NETs. Confocal microscopy imaging of fixed samples. Cells were kept in RPMIcomp. + 10 mM HEPES.

For the initial experiments in Figure 1, broad-spectrum UVA (300–400 nm) light was used, and cells were observed over 3–3.5 h with a combination of continuous and intermittent light exposure during live-cell imaging. To verify the obtained results in a more controlled fashion, we established a precisely defined LED-light-based setup and irradiated the cells from below with light of distinct wavelengths and doses (Figure 2). Cells were exposed to 3.5, 18, 35, or 70 J/cm^2^ of UVA light (375 nm) and 21, 54, 107, or 214 J/cm^2^ of visible blue light (470 nm). The LED-light clearly induced chromatin decondensation dose-dependently starting with significant rates of NETosis at 70 J/cm^2^ for 375 nm and at 107 J/cm^2^ for 470 nm, respectively (Figures 2A,B). Interestingly, the morphology of NETs induced by LED-light slightly differed from PMA-induced NETs. Light-induced NETs, as well as the remaining cell body, appeared smaller compared to NETs stimulated with PMA. These differences possibly originate from the strong PMA-induced cell adhesion, which typically occurs in early stages of NETosis. For both tested wavelengths, the decondensed chromatin colocalized with MPO, a typical feature of NET formation (Figure 2C). Additionally, a clear citrullination of histone 3 (H3Cit) could be observed. This citrullination typically appeared during early stages of chromatin decondensation whereas MPO seemed to colocalize more with strongly decondensed chromatin and was especially prominent in the released NET fibers (Figure 2C).

### Light-Induced NETosis Depends on MPO and NE

One of the hallmarks of NET formation is the strong dependency on enzyme activity, especially in the first phase of NETosis, enabling histone modification and, consequently, chromatin decondensation (17). The involved enzymes can vary among different stimuli. In most cases, the activation of granular enzymes such as NE and MPO or members of the PAD family, particularly PAD4, are indispensable (11). Therefore, we inhibited the activity of various enzymes known to be involved in chromatin decondensation or that are required for associated signaling cascades of well-described activators of NETosis.

**Figure 3:**
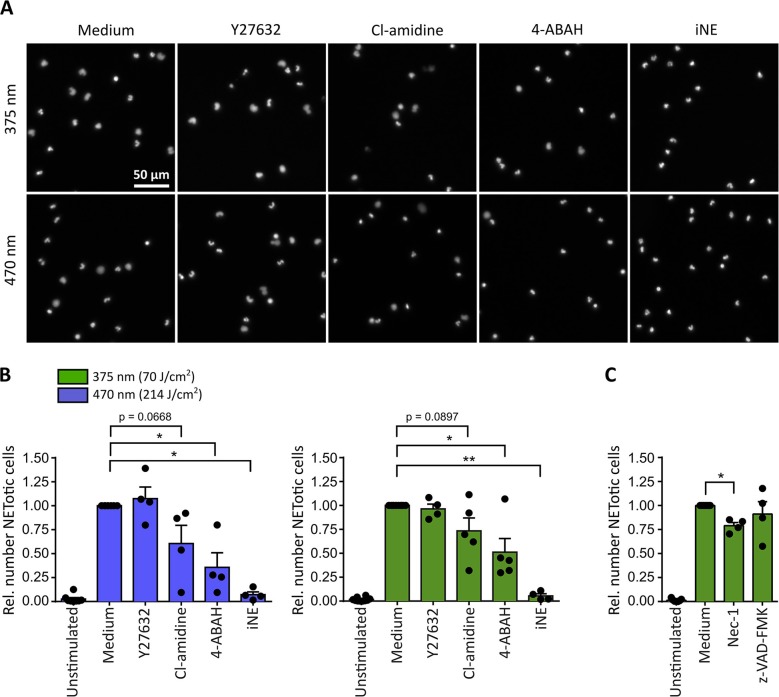
Neutrophil elastase (NE) and myeloperoxidase (MPO) are indispensable for light-induced NETosis. **(A)** Representative images of neutrophil nuclei stained by Hoechst after exposure to UVA (70 J/cm^2^ of 375 nm) or blue light (214 J/cm^2^ of 470 nm) in the presence of specific inhibitors. NETosis is clearly reduced upon inhibition of MPO (4-ABAH, 100 μM), NE (GW-311616A, iNE, 5 μM) and, to a lower extent, of PAD enzymes (Cl-amidine, 200 μM). However, neutrophils are still able to undergo NETosis in the presence of ROCK 1/2 inhibition by Y-27632 (20 μM). **(B)** Quantification of NETotic cells after irradiation with LED-light of 375 or 470 nm, respectively, in the presence of Y-27632, Cl-amidine, 4-ABAH, or iNE. The inhibition of MPO and NE significantly reduces light-induced NET formation. Inhibition of PAD-enzymes decreases the number of NETotic cells induced by LED-light. However, NETosis appears independent of ROCK 1/2 activity. Statistics: two-tailed paired *t*-test. ^*^p < 0.05, ^**^p < 0.01. N = 4–5 independent experiments. Error bars = SEM. Cells kept in RPMIcomp. + 10 mM HEPES. **(C)** Light-induced NETosis (70 J/cm^2^ of 375 nm) occurs independently of z-VAD-FMK (20 μM, pan-caspase inhibitor) and is reduced by Nec-1 (50 μM, RIP1 kinase inhibition). Statistics: two-tailed paired *t*-test. ^*^p < 0.05. *N* = 4 independent experiments. Error bars = SEM. Cells were kept in RPMIcomp. + 10 mM HEPES.

For both tested wavelengths a significant reduction of NETosis was observed in the presence of the MPO-inhibitor 4-aminobenzoic acid hydrazide (4-ABAH, 100 μM) (48) or the NE-inhibitor GW-311616A (iNE, 5 μM) (49) (Figures 3A,B). Both inhibitors efficiently blocked the decondensation of chromatin (Figure 3A), thus indicating that decondensation in light-induced NETosis depended on MPO and NE activity as reported for PMA-induced NETosis (18, 19). Additionally, inhibition of PAD activity by Cl-amidine (200 μM) (50) reduced NET formation after irradiation with light of both wavelengths by around 25-50% (Figure 3B). Therefore, it is likely that the activity of PAD enzymes can enhance light-induced NETosis by modifying proteins, particularly histones, by citrullination (23, 51, 52). Dependency on Rho-associated coil kinase 1 and 2 (ROCK 1/2) activity, which is implicated in cytoskeleton regulation, has only recently been linked to PMA-induced NETosis (17). Nonetheless, irradiation of neutrophils in the presence of Y-27632 (20 μM) blocking the ATP binding site of ROCK 1/2 (53), showed no effect on NETosis rates in response to light (Figure 3B). It is important to note that inhibitors were still functional after irradiation with UVA light as demonstrated in PMA-induced NET formation (Supplementary Figure 2).

In order to exclude that the observed effects were associated with neutrophil apoptosis, the involvement of caspases after UVA irradiation was investigated. To this end, cells were irradiated in the presence of the pan-caspase inhibitor z-VAD-FMK (20 μM) (54). Indeed, the involvement of apoptotic pathways was not detected. In contrast, a contribution of the receptor-interacting protein kinase (RIPK) 1/3-mixed lineage kinase domain-like protein (MLKL)-necroptosis-pathway, which has been reported for instance in PMA-induced NETosis (55), could not be excluded. Inhibition by the RIP1 kinase inhibitor necrostatin-1 (Nec-1, 50 μM) (56) decreased the rate of NETotic cells by ~25% (Figure 3C).

Altogether, these findings suggest that UVA and blue light induce NETosis in an MPO- and NE-dependent manner. This process can be supported by PAD enzymes and appears to be independent of caspase activity.

### Light-Induced NETosis Is Mediated by Riboflavin Excitation and Subsequent ROS Generation

**Figure 4:**
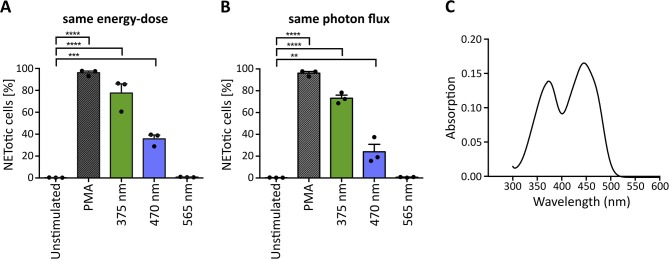
Wavelength-dependency of NET formation correlates with light absorption of riboflavin. UVA and blue light significantly induce NET formation at **(A)** the same energy-dose and **(B)** the same photon flux, whereas green light (565 nm) does not induce NETosis under either condition. Statistics: repeated measure one-way-ANOVA with Bonferroni's multiple comparisons test (tested against unstimulated cells). ^**^p < 0.01, ^***^p < 0.001, ^****^p < 0.0001. *N* = 3 independent experiments. Error bars = SEM. Cells kept in RPMIcomp. + 10 mM HEPES. **(C)** Riboflavin absorbs light in the UVA-blue light region with maxima at 373 and 445 nm. At 375 nm, the absorption is slightly higher (0.138), than at 470 nm (0.130).

The penetration of light through the human skin depends strongly on the wavelength. Approximately 10–15% of UVA light and 40–50% of blue light can pass the epidermis and reach deeper layers (33, 34). In principle, light of higher wavelengths penetrates deeper into the skin (33). To evaluate whether light of higher wavelengths is also sufficient to induce NET formation, we irradiated neutrophils with light up to 700 nm (565 nm LED; green light), of which more than 60% reach the dermis and in part, even the subcutaneous tissue (34, 57). To make comparisons possible, cells were irradiated with the same light energy-dose (Figure 4A) and the same photon flux for each wavelength (Figure 4B). The calculations were based on 70 J/cm^2^ 375 nm-LED light. Interestingly, at the same energy-dose and same photon flux, neutrophils did not undergo NETosis after exposure to green light, whereas irradiation with UVA or blue light revealed robust NETosis (Figures 4A,B). However, irradiation with 375 nm induced higher rates of NETotic cells compared to 470 nm. Possible explanations for this phenomenon include the slightly higher absorption of riboflavin at 375 nm (0.138 vs. 0.130) as well as the higher energy of the photons at 375 nm, leading to higher energy within the riboflavin molecule and subsequent excitation.

Several substances, which are present at high concentrations in human skin, can absorb light in the UV-VIS region and were reported to enhance light-mediated tissue damage. One of the most prominent and well-documented photosensitizer is riboflavin (38, 58). Riboflavin is present in tissues with permanent light exposure such as the skin and eyes [3 and 1.7 mg/kg dry matter, respectively (38)] and has multiple essential biological functions. Most prominently, it acts as a precursor for FAD and FMN in flavoprotein-dependent processes (58, 59). Photosensitizing mechanisms of riboflavin are based on the absorption of UVA and blue light with maxima at 373 and 445 nm as confirmed within this study in line with previously published data (60) (Figure 4C). Riboflavin is excited to a stable triplet-state *via* a short-lived singlet-state. The excited triplet-state can directly react with oxygen (type II photoreaction) or with reactive substrates (type I photoreaction) to radicals or radical anions. These radicals can then further react with molecular oxygen to hydrogen peroxide (H_2_O_2_) or hydroxyl radicals *via* superoxide anion radicals (38, 61, 62) (for a potential type I photoreaction with tryptophan see Supplementary Figure 3). Importantly, riboflavin is present in the culture medium RPMI at 0.2 mg/l and was previously linked to an increased phototoxicity in culture media in *in vitro* studies. Such reactions were observed for instance in combination with the culture buffer 4-(2-hydroxyethyl)-1-piperazineethanesulfonic acid (HEPES) (63) or amino acids like tryptophan and tyrosine supplemented to RPMI (64).

**Figure 5:**
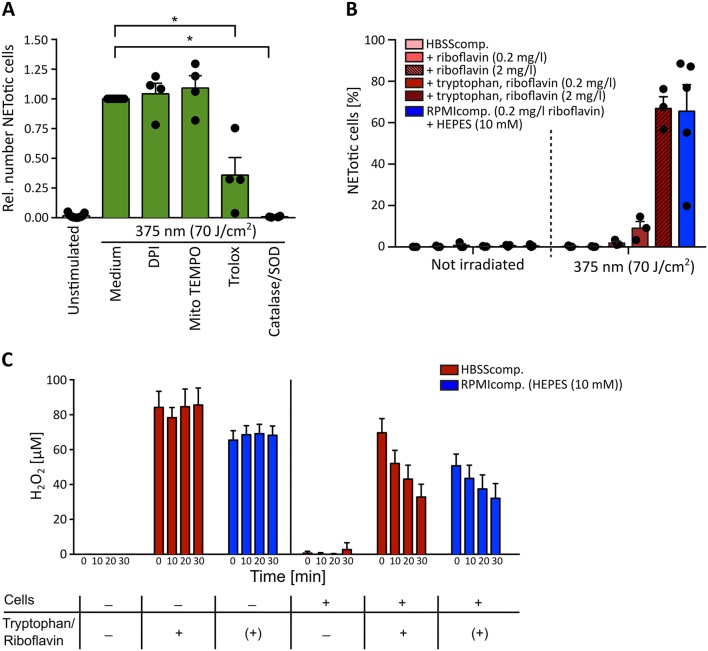
Light-induced NETosis depends on extracellular reactive oxygen species (ROS) generation. **(A)** Light-induced NETosis is significantly inhibited by the ROS scavenger Trolox (50 μM) and by exclusively scavenging external ROS by a combination of catalase/SOD (2,000 and 50 U/ml). NETosis appears to be independent of intracellular ROS production by NADPH oxidase, as shown by inhibition with DPI (1 μM) or mitochondrial ROS, inhibited by MitoTEMPO (5 μM). Statistics: two-tailed paired *t*-test. ^*^p < 0.05. *N* = 4 independent experiments. Error bars = SEM. Cells were kept in RPMIcomp. + 10 mM HEPES. **(B)** 70 J/cm^2^ of 375 nm light clearly induces NET formation in HBSScomp. + riboflavin (2 mg/l) + tryptophan (1 mM). The obtained NET rates are comparable with NETosis induced in RPMIcomp. (containing 0.2 mg/l riboflavin) + 10 mM HEPES. In contrast, irradiation in HBSScomp. alone or supplemented only with riboflavin (0.2 or 2 mg/l) does not lead to NET formation. *N* = 3–5 independent experiments. Error bars = SEM. **(C)** Induction of extracellular H_2_O_2_ levels by light measured by AmplexRed. Irradiation of HBSScomp. (red) + 2 mg/l riboflavin + 1 mM tryptophan or RPMIcomp. (0.2 mg/l riboflavin, 0.024 mM tryptophan) + 10 mM HEPES (blue) induces stable extracellular H_2_O_2_ levels between 60 and 100 μM. Similar H_2_O_2_ levels are measurable after irradiation of neutrophils in these two media. In the presence of neutrophils, the H_2_O_2_ levels are continuously reduced over 30 min to around 30–40 μM. In HBSScomp. without riboflavin and tryptophan, neither direct irradiation nor irradiation in the presence of neutrophils causes any increase in H_2_O_2_ levels. “+” = addition of 2 mg/l riboflavin and 1 mM tryptophan. “(+)” = 0.2 mg/l riboflavin and 0.024 mM tryptophan within RPMI. *N* = 3–4 independent experiments. Error bars = SEM.

Given the wavelength-dependency of NETosis, the absorption spectra of riboflavin (Figure 4) and the fact that H_2_O_2_ as a ROS can induce NETosis (16, 65–67), it appeared likely that the observed light-induced NETosis was mediated by the excitation of riboflavin and subsequent ROS production. To test this hypothesis, we first scavenged ROS by the cell-permeable vitamin E derivate Trolox at a concentration of 50 μM (68). Indeed, scavenging ROS with Trolox significantly reduced NET formation in response to UVA irradiation (Figure 5A). To further analyze this mechanism, extra- and intracellular ROS generation were addressed separately. Extracellular ROS was scavenged by a mixture of catalase and superoxide dismutase (SOD) (2,000 U/ml and 50 U/ml, respectively) (69, 70), NADPH oxidase-derived ROS was blocked by diphenyleneiodonium chloride (DPI, 1 μM) (71) and mitochondrial-derived ROS was inhibited by MitoTEMPO (5 μM) (72). While the inhibition of NADPH oxidase- and mitochondrial ROS generation showed no effect on the obtained NETosis rates, scavenging extracellular ROS by catalase and SOD abrogated NETosis completely (Figure 5A). This result strongly supported the hypothesis that extracellular substrate-mediated production of ROS facilitated light-induced NETosis. Furthermore, the addition of the catalase-SOD-mixture after the irradiation was still fully sufficient to inhibit NETosis (Supplementary Figure 4A) excluding the fact that side products of catalase or SOD themselves induced by UVA irradiation were responsible for the observed effect. These results also highlight the differences between the here-studied light-induced NET formation and the “classical” PMA-induced NETosis. In contrast to light-induced NETosis, the formation of NETs in response to PMA depended on the activity of the NADPH-oxidase, which is in agreement with several previous reports (16) and was independent of mitochondrial ROS generation. Additionally, we observed a decreased NETosis rate following PMA stimulation after scavenging extracellular ROS and/or intracellular H_2_O_2_ (Supplementary Figure 4B). However, H_2_O_2_ scavenging was not sufficient to completely block NETosis. Therefore, especially the activity of NADPH oxidase and the subsequent ROS-mediated signaling appears to be a requisite for PMA-induced NETosis.

In a second step, we studied the mechanism of extracellular ROS generation in greater depth. As described above, aromatic amino acids such as tryptophan can react with excited riboflavin/flavoproteins. As essential components of proteins, these amino acids are frequently expressed in human skin and can, therefore, contribute to ROS formation triggered by riboflavin excitation. To precisely evaluate the contribution of tryptophan to NET formation, neutrophils were irradiated in the culture buffer HBSS containing 0.5% FCS (HBSScomp.) and supplemented with 0.2 or 2 mg/l riboflavin, a reasonable physiological range of riboflavin within human skin (38), in the presence or absence of 1 mM tryptophan. Indeed, in comparison to non-irradiated cells, UVA light exposure resulted in marked NETosis in the presence of both riboflavin and tryptophan. This effect was increased with the higher riboflavin concentration. In contrast, no NET formation was observed in pure HBSScomp. or in buffer supplemented exclusively with riboflavin (Figure 5B). These results strongly support the hypothesis that riboflavin mediated NET formation increases in the presence of additional substrates like tryptophan. Interestingly, a similar effect was observed for HEPES-buffer in RPMIcomp. NET rates markedly increased in the presence of HEPES, the standard medium condition used for this study, compared to RPMIcomp. without HEPES (Supplementary Figure 5A).

To confirm this hypothesis, extracellular ROS levels were measured after irradiation in the above-described solutions in an AmplexRed assay. In line with the observed NET rates (Figure 5B), irradiation with UVA light led to dramatically increased extracellular H_2_O_2_ levels in both RPMIcomp. + HEPES as well as HBSScomp. + riboflavin (2 mg/l) + tryptophan (Figure 5C). Sole irradiation of these two media compositions consistently resulted in stable H_2_O_2_ levels of 60–100 μM. In contrast, irradiation of supplement-free HBSScomp. as well as culturing of cells in media without irradiation did not lead to H_2_O_2_ production (Figure 5C; Supplementary Figure 5B). As a control, extracellular H_2_O_2_ levels were determined after activation with 100 nM PMA. As depicted, lower levels of extracellular H_2_O_2_ were observable after PMA treatment, which increased over time (Supplementary Figure 5B) (70). Importantly, in this experimental setup, we obtained NET rates comparable to the rates displayed in Figure 5B after UVA irradiation of cells in RPMIcomp. + HEPES or HBSScomp. + riboflavin (2 mg/l) + tryptophan (Supplementary Figure 5C). Overall, these results support the hypothesis that ROS-mediated UVA/blue light-induced NET formation is a consequence of riboflavin excitation. Our findings indicate that excited riboflavin reacts with biological substrates such as tryptophan and thus causes ROS production.

## Discussion

UVA and blue light penetrate human skin, and the energy-rich UV light in particular, can cause severe tissue injury *via* immunological and inflammatory effects (73). Both UVA and blue light from the sun reach the earth's surface and penetrate the human epidermis.

**Figure 6:**
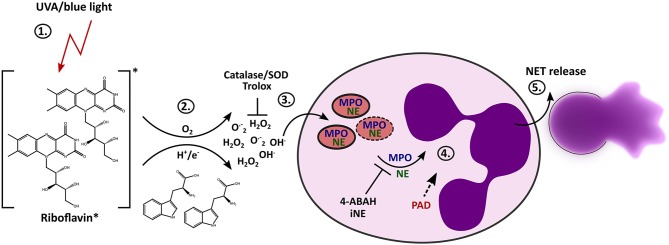
Proposed mechanism of light-induced NETosis. (1) Riboflavin absorbs UVA and blue light and is excited to the triplet state. (2) Tryptophan or other substrates (e.g., HEPES-buffer, tyrosine) transfer electrons or protons to triplet-riboflavin, which leads to ROS production. (3) Extracellular ROS generation (e.g., H_2_O_2_/OH^·^ or O^.−^_2_) by excited riboflavin in the presence of reactive substrates is required for further progression of NET formation. Extracellular ROS is scavenged by Trolox or catalase/SOD. (4) Extracellular ROS most likely activates the translocation of granular enzymes like NE and MPO to the nucleus, which leads to the decondensation of chromatin. The activation of NE and MPO is mandatory for chromatin decondensation and can be inhibited by 4-ABAH and iNE. Citrullination by PAD enzymes can presumably enhance decondensation. (5) After full decondensation, cells release the NET.

It has been shown that *in vitro* irradiation of neutrophils with UVB (40) or UVC light (74) can cause cell death and that exposure to UVA light can enhance ROS production (75). Activation of NETosis has also been considered in response to UVC light (41) However, the exact mechanisms behind it and the influence of blue and long-wave UV light on NET formation remains enigmatic. We have discovered that light of the UVA or blue spectrum is able to induce the release of NETs. The formation of NETs in this scenario depends on extracellular ROS, which are generated through the excitation of riboflavin in conjunction with substrates such as tryptophan. During the “classical” NETosis cascade, NE and MPO are typically released from neutrophil granules and translocate to the nucleus where they promote chromatin decondensation. According to our findings, light-induced NET formation also depends on MPO and NE. Additionally, inhibition of PAD activity by Cl-amidine during irradiation with UVA or blue light clearly showed reduction in NET formation, and histones were citrullinated after light irradiation with both wavelengths. Therefore, the citrullination of histones most likely contributes to decondensation of chromatin in this setting. Altogether, these results imply that this novel light-mediated NET formation culminates in the initiation of the “classical” pathway of suicidal NETosis by engaging the enzymes MPO, NE and PAD4 (Figure 6), as extensively described for different stimuli throughout the last years (11).

Interestingly, in response to near infrared (980 nm) laser light, a ROS-dependent form of NET formation has recently been reported (76), which was shown to involve autophagy signaling. In spite of the fact that NETs have been shown to impair wound healing (43), this laser light-mediated NET formation was suggested to be particularly relevant in the context of photobiostimulation with 980 nm diode lasers used to improve wound healing (76). However, the exact mode of action of the laser light remains unclear. The question whether autophagy is implicated in the here-described mechanism of light-induced NETosis warrants further studies.

In a different study, UVC light was reported to trigger apoptosis and/or NOX-independent suicidal NETosis in a dose-dependent manner (41). Highly energetic UVC light had triggered NETosis dependent on mitochondrial ROS and p38 MAPK activation. Additionally, certain biochemical features of apoptosis accompanied UVC-dependent NETosis. Therefore, the authors termed this new NETosis pathway “ApoNETosis” (41). Of note, one must bear in mind that naturally occurring UVC light is almost completely absorbed by the ozone-layer (31) and thus does not reach the human skin. Moreover, while artificial light sources are able to generate UVC light, this would not be expected to penetrate in high amounts across the stratum corneum of the skin. Thus, the physiological relevance of UVC light in the context of NETosis remains disputable. Nevertheless, there may be some relevance for *ex vivo* neutrophil studies.

The mechanism we described here is NOX-independent, similarly to “ApoNETosis.” However, UVA or blue light-induced NETosis does not involve mitochondrial ROS production, but clearly depends on MPO and NE activation (Figures 3, 5A). The release of NE from the azurosome depends on MPO activity and NADPH-dependent ROS generation (19). In this study, we detected a high extracellular light-induced ROS production in riboflavin-containing media, which was stable for over 30 min after irradiation (Figure 5C). This result indicates that riboflavin excitation together with reactive substrates induces high levels of stable ROS species like H_2_O_2_ (77). H_2_O_2_ can easily diffuse into the cell and could therefore directly activate the release of serine proteases from the neutrophilic granules. Thus, in NETosis induced by extracellular ROS, additional production of ROS via NADPH-oxidase would not be required, which explains our observation that light-induced NETosis was independent of NADPH.

Importantly, the production of H_2_O_2_ in these media (especially RPMI + HEPES) and the subsequent release of NETs has to be considered for live cell imaging of neutrophils, especially when working with light of the blue or UVA spectrum. Choosing media without light-sensitive substances would avoid light-induced, ROS-mediated NETosis *ex vivo*.

Typically, in non-inflamed skin, neutrophils closest to the skin surface are found within the superficial arterio-venous plexus of the papillary dermis (57). The transmission of light depends on the local skin composition, but one can estimate that 10–15% of UVA light and 40–50% of blue light reach the upper papillary dermis (33, 34).

In our study, we observed NET formation after irradiation of neutrophils starting with 18 J/cm^2^ of 375 nm and 54 J/cm^2^ of 470 nm LED-light (Figure 2). These doses correspond to around 40 h (375 nm) or 9 h (470 nm LED spectrum) sun exposure at the specific wavelength, respectively. However, under real-life conditions one must consider the complex spectrum of sunlight, which is not limited to single wavelengths, as well as factors such as the thickness of the local ozone-layer, location on earth, weather, irradiation angle, level above the sea as well as time of day and season which will affect light penetration (31). Also, UVA and blue light-induced NET formation may play a more important role in pathological situations of skin if patients suffer from a generally heightened propensity for light-induced NET formation. Additionally, neutrophils may be more strongly affected by light, for example if they are present in higher layers of the skin under inflammatory conditions. Indeed, inflammatory skin diseases are often associated with enhanced vasodilation, perivascular inflammatory infiltrates and even neo-angiogenesis. In many cases, this is associated with an enhanced infiltration of neutrophils and other inflammatory cells into the epidermis, which would again facilitate light-induced NET formation. Additionally, exposure to UV-Vis light particularly of the UVB region appears to directly recruit neutrophils into upper layers of the skin (39). This phenomenon has been proposed to be mediated for instance by the production of cytokines like IL-8 or TNFα by other cell types such as keratinocytes and fibroblasts in response to UVB irradiation (78, 79).

In healthy skin, ROS levels are closely regulated by enzymatic and non-enzymatic antioxidant like glutathione-peroxidase and catalase, vitamin C, and vitamin E. As human skin contains significant amounts of riboflavin, ROS generation by excitation of riboflavin and subsequent reaction with active substrates (e.g., in a type I photoreaction) including aromatic amino acids is very likely (38, 62, 64, 80, 81). Nonetheless, the antioxidants systems mentioned above balance out these reactions under normal circumstances. In the context of diseases, this complex antioxidant system can be dysregulated or exhausted. For instance, extensive or continuous exposure of sunlight causes increased ROS levels with associated inflammation and tissue damage, even in healthy individuals (82). This ROS-associated oxidative tissue damage is mostly mediated by the deeper-penetrating UVA portion of the spectrum.

Interestingly, increased redox stress has been documented in autoimmune disorders (83). For instance, SLE appears to be frequently associated with high oxidative stress indicated by decrease in antioxidant systems, general increased ROS levels as well as elevated antibodies against oxidatively modified proteins (84). In fact, neutrophils from SLE patients reveal higher oxidative burst (85) with decreased intracellular antioxidant systems (86), what makes them less resistant toward extracellular ROS generation.

Additionally, neutrophils of patients suffering from psoriasis and SLE were reported to be generally primed for NETosis (5, 87) and NETosis is clearly involved in the pathogenesis of many chronic inflammatory and autoimmune diseases (2). For example, autoantigens against NET components were detected in SLE (88). Furthermore, impaired clearance of NETs has been described in the pathogenesis of SLE, leading to an accumulation of potential autoantigens in the form of NET components (89).

Most likely, both conditions–ROS imbalance and an increased propensity for NET formation–will add to one another and result in a ROS-mediated inflammatory loop. To what extent NETosis induced by light contributes to this cycle, has to be investigated within *in vivo* studies.

Several autoimmune diseases, most prominently the above-mentioned systemic and cutaneous lupus erythematosus as well as dermatomyositis, show severe photosensitivity. Patients suffering from lupus, whether acute, subacute, or chronic, develop new cutaneous lesions after sun exposure. Even an exacerbation of SLE like fatigue or joint pain has been well-documented after light-exposure (28). Nevertheless, the exact pathophysiological mechanism has not been unraveled yet and involves a differential interplay between different light-induced effects (28).

Most likely, the abnormal response of lupus patients to light is not a monocausal one but given the importance of NETs in this disease and other autoimmune disorders it appears likely that a connection between light exposure and NET formation is an important factor. The question of whether neutrophils of lupus and dermatomyositis patients are *per se* more prone to light-induced NETosis and whether these reactions can be prevented warrant further translational studies. In this context, the question as to what extent light-induced NETs are targets for autoantibody formation, also remains a highly interesting one.

Altogether, it is important to bear in mind that, according to our calculations, NET formation in response to moderate doses of light is most likely not a frequent event and that in healthy individuals NETs are usually rapidly cleared by DNases and subsequent phagocytosis (90, 91). Thus, it is unlikely that exposure to modest doses of sunlight in healthy individuals will lead to a profound inflammatory reaction. However, the here-presented mechanism could be highly important in pathological conditions, such as those discussed above.

On the other hand, light-mediated, ROS-dependent NETosis may also be instrumentalized in a clinical setting to help fight bacteria in bacterial keratitis, as a recent study has impressively shown. Here, the combination of UVA light with riboflavin was used in the “photochemical” therapy of bacterial keratitis due to its bactericidal effect (92). Furthermore, one may speculate whether at least a part of the effect of photodynamic therapy (PDT), can be explained by NET generation. Visible light irradiation, especially of the red but also blue light spectrum, is used in combination with photosensitizers such as aminolevulinic acid (ALA) or methyl aminolevulinate (MAL) in the therapy of basal cell carcinomas, Bowen's disease, and actinic keratosis (*carcinoma in situ*) (93). This therapy is based on the excitation of the photosensitizer porphyrin originating from ALA or MAL and subsequent ROS generation similar to what we have reported in this study for riboflavin. Therefore, the highly interesting question remains whether NETosis can also be activated by ROS generation in this therapeutic context.

In conclusion, we show that UV-Vis light causes ROS-dependent NET formation, which most likely bears great clinical relevance for important diseases such as SLE and dermatomyositis.

## Data Availability Statement

The datasets generated for this study are available on request to the corresponding author.

## Ethics Statement

The studies involving human participants were reviewed and approved by Ethics Committee of the University Medical Center (UMG) Göttingen. The patients/participants provided their written informed consent to participate in this study.

## Author Contributions

EN and KB performed most experiments, co-wrote the paper, and designed the study together with LE. LE supervised the project. JB provided selected data sets. SK, MS, and IB provided crucial scientific input as well as technical support. All authors proofread and edited the final manuscript.

### Conflict of Interest

The authors declare that the research was conducted in the absence of any commercial or financial relationships that could be construed as a potential conflict of interest.
