# Supplementary material for: Blue and Long-Wave Ultraviolet Light Induce in vitro Neutrophil Extracellular Trap (NET) Formation
Source: Front Immunol. 2019 Oct 25;10:2428. doi: 10.3389/fimmu.2019.02428 (PMC6823194; doi:10.3389/fimmu.2019.02428)
Supplement: Supplementary file 2 [file Data_Sheet_1.docx]

**Supplementary material**

**Blue and Long-Wave Ultraviolet Light Induce *in vitro* Neutrophil Extracellular Trap (NET) formation**

Elsa Neubert^1,2*^, Katharina Marie Bach^1*^, Julia Busse^1^, Ivan Bogeski^3^,

Michael P. Schön^1,5^, Sebastian Kruss^2,4^ and Luise Erpenbeck^1+^

^1^Department of Dermatology, Venereology and Allergology, University Medical Center Göttingen, Göttingen, Germany

^2^Institute of Physical Chemistry, Göttingen University, Göttingen, Germany

^3^[Institute of Cardiovascular Physiology](http://www.med.uni-goettingen.de/de/content/ueberuns/218_380.html), University Medical Center Göttingen, Göttingen, Germany

^4^Center for Nanoscale Microscopy and Molecular Physiology of the Brain (CNMPB), Göttingen, Germany

^5^Lower Saxony Institute of Occupational Dermatology, University Medical Center Göttingen and University of Osnabrück, Germany

**^*^**E.N. and K.M.B. contributed equally to this study.

^+^Correspondence should be addressed to L.E. (email: [luise.erpenbeck@med.uni-goettingen.de](mailto:luise.erpenbeck@med.uni-goettingen.de))

**Supplementary Movie**

Changes of the nuclear morphology of human neutrophilic granulocytes during UVA-induced NETosis. Staining: Hoechst.

**Supplementary Figures**


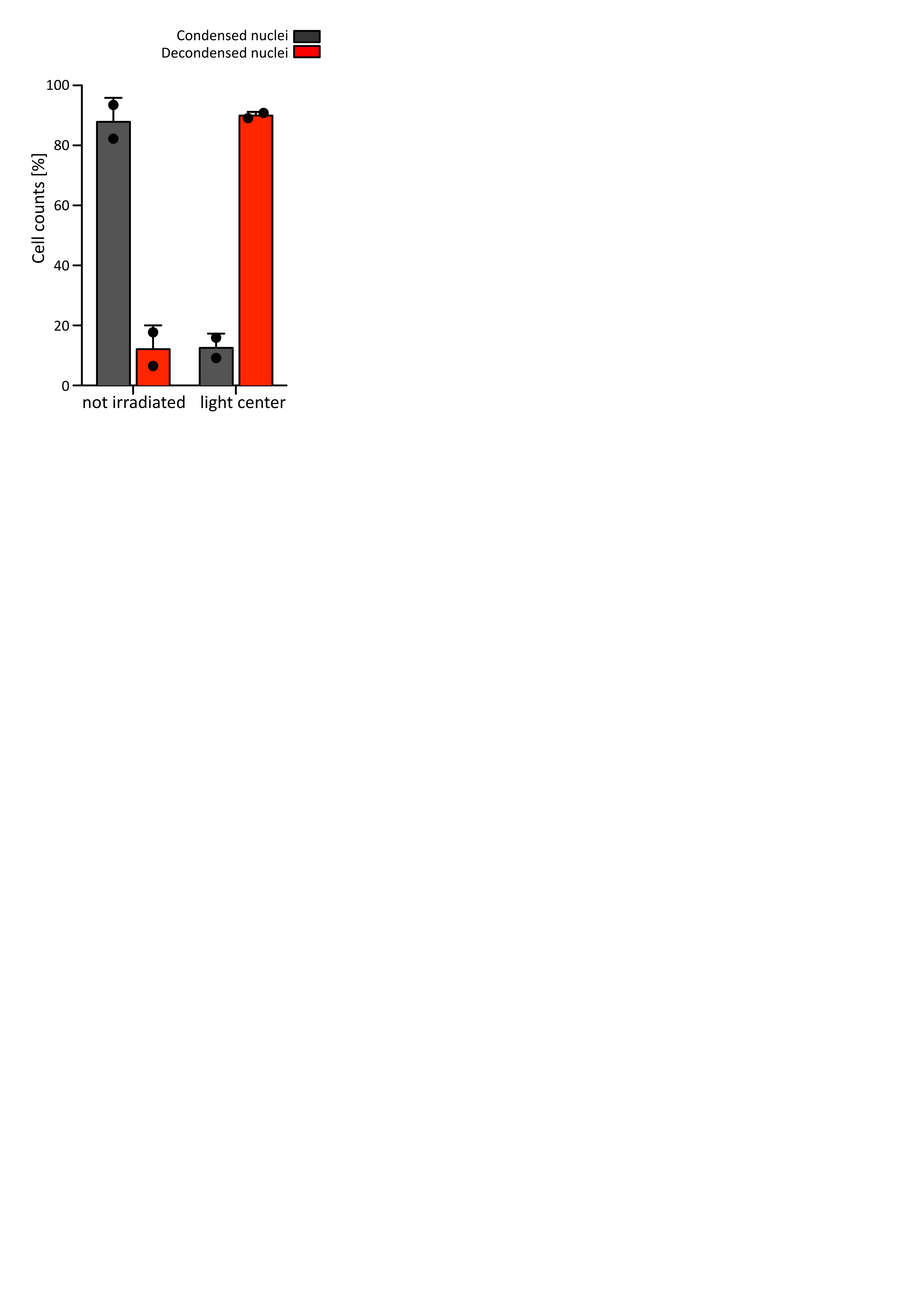


**Supplementary Figure 1: Quantification of locally-restricted light-induced NETosis.** NETosis rates are 60-70% higher in the center of light exposure compared to non-illuminated areas. N = 2 independent experiments. Error bars = SD.

**
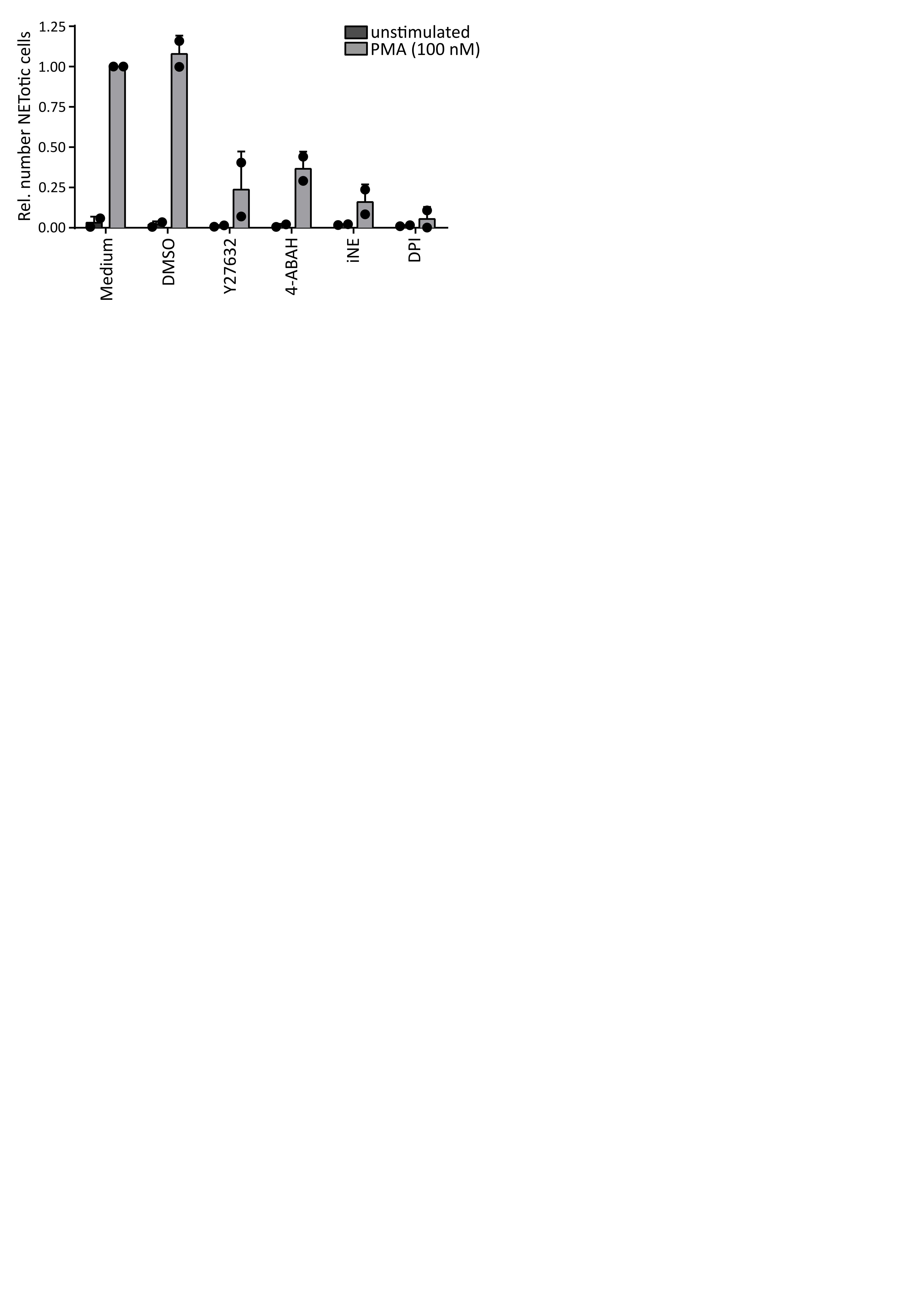
**

**Supplementary Figure 2: Functionality of NET inhibitors.** NETosis in response to PMA (100 nM) is inhibited by 4-ABAH (100 µM), iNE (5 µM), DPI (1 µM) and Y-27632 (20 µM), even after inhibitors were treated with 70 J/cm^2^ of 375 nm, ruling out that inhibitor function was altered by light exposure. Induction of NETosis by PMA is not altered upon the addition of DMSO (solvent for inhibitors) in the highest concentration used (1%). Cells were kept in RPMIcomp. + 10 mM HEPES. N = 2 independent experiments. Error bars = SD.


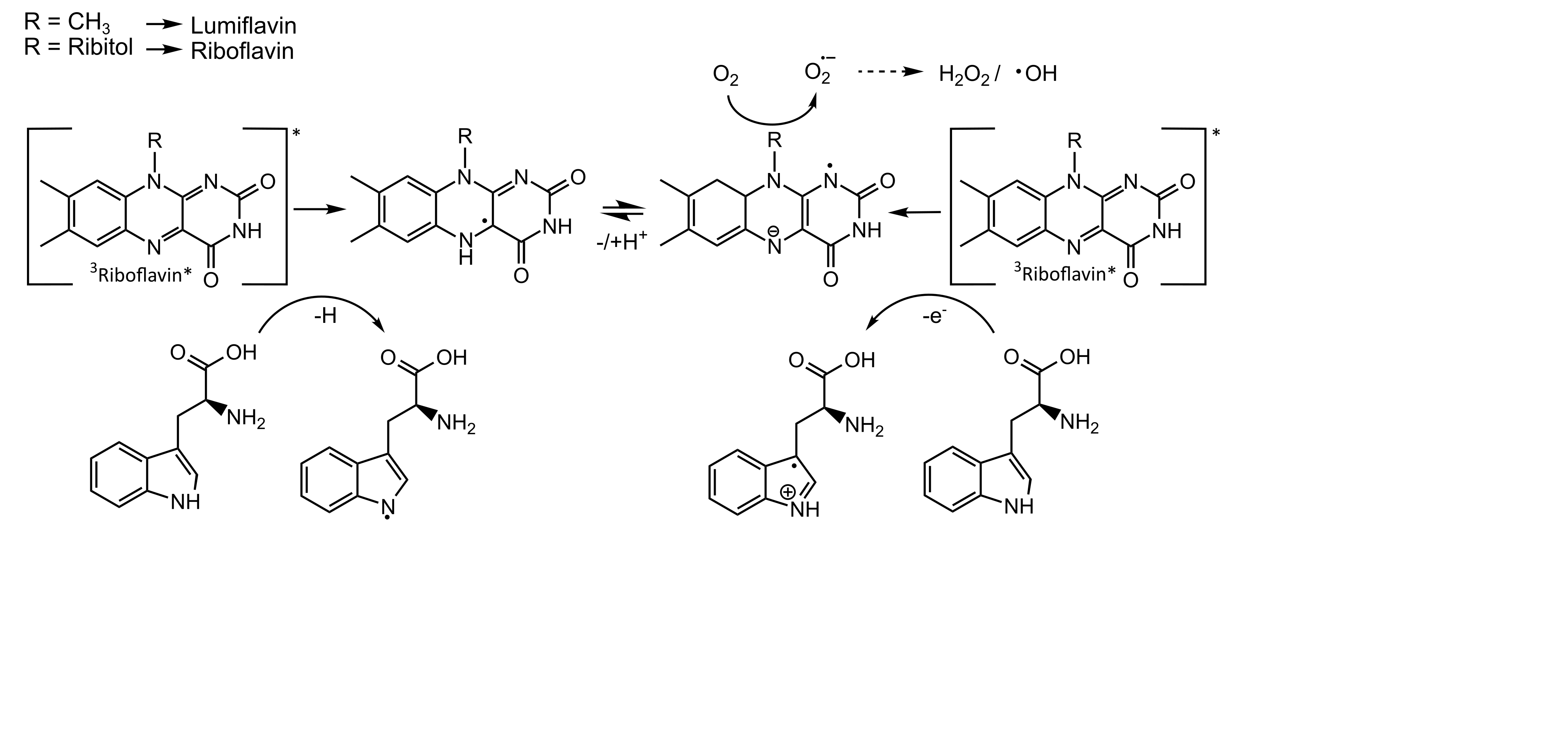


Supplementary Figure 3. Reaction of triplet-riboflavin with tryptophan. Tryptophan can transfer an electron or a proton to excited triplet-riboflavin (type I photoreaction). This intermediate can further react with molecular oxygen and thus generate ROS. Riboflavin is transferred back to the ground state. This schema is based on previous published reactions between riboflavin and tryptophan[^1-4^](#_ENREF_1).


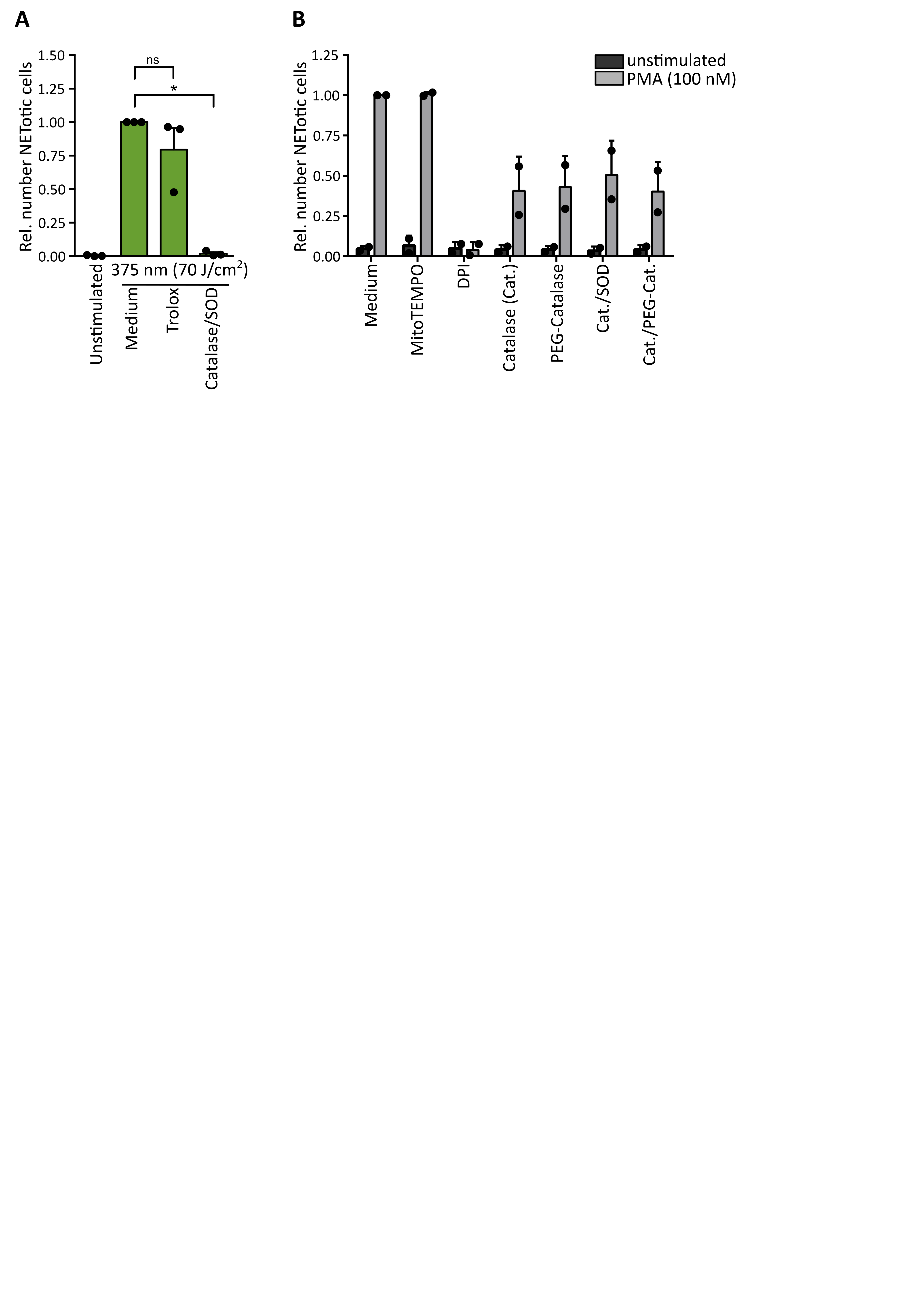


**Supplementary Figure 4**. **ROS dependency of light- and PMA-induced NETosis.** (**A**) Catalase/SOD significantly inhibits NETosis and Trolox (50 µM) clearly reduces NET rates, when added after full illumination with 70 J/cm^2^ of 375 nm. Cells were kept in RPMIcomp. + 10 mM HEPES. N = 3 independent experiments. Error bars = SEM. Statistics: two-tailed paired *t*-test. *p < 0.05. ns = not significant. (**B**) Inhibition of NADPH oxidase activity by DPI (1 µM) shows clear reduction of NETosis induced by PMA (100 nM) whereas the inhibition of mitochondrial ROS production by MitoTEMPO (5 µM) has no impact on PMA-induced NETosis. Addition of catalase (2,000 U/ml), PEG-catalase (2,000 U/ml), catalase/SOD (2,000 U/ml, 50 U/ml) or of a mixture of catalase (1,000 U/ml) and PEG-catalase (1,000 U/ml) reduce PMA-induced NETosis by around 30-50%. Cells were kept in RPMIcomp. + 10 mM HEPES. N = 2 independent experiments. Error bars = SD.


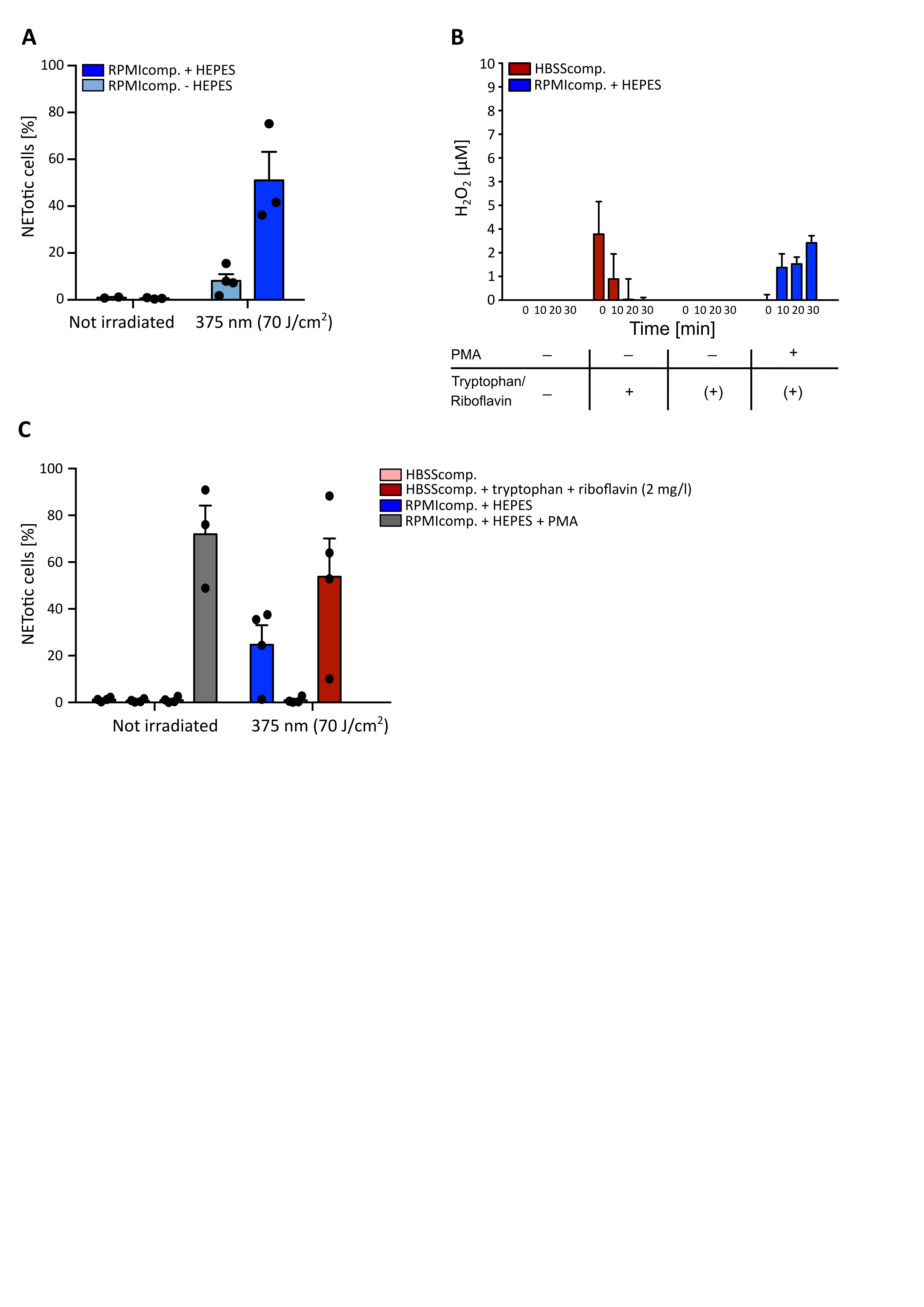


Supplementary Figure 5. Light-induced NETosis is dependent on extracellular ROS. (A) NET rates in response to light of 375 nm 70 J/cm^2^ are enhanced in the presence of the culture buffer HEPES (10 mM). N = 3-4 independent experiments. Error bars = SEM. (B) Without irradiation, H_2_O_2_ levels are stable with and without addition of riboflavin and tryptophan. After stimulation with 100 nM PMA extracellular H_2_O_2_ increase within 30 min up to 3 µM. “+” = addition of 2 mg/l riboflavin and 1 mM tryptophan. “(+)” = 0.2 mg/l riboflavin and 0.024 mM tryptophan within RPMI. N = 3-4 independent experiments. Error bars = SEM. (C) During AmplexRed measurements NETosis is stably induced by PMA (100 nM) and light in RPMIcomp. with HEPES (10 mM) and HBSScomp. supplemented with tryptophan (1 mM) and riboflavin (2 mg/l). N = 3-4 independent experiments. Error bars = SEM.

**References**

1. Silva E, Ugarte R, Andrade A, Edwards AM. Riboflavin-sensitized photoprocesses of tryptophan. *J Photochem Photobiol B*. 1994;23(1):43-48.

2. Shen L, Ji HF. A theoretical study on the quenching mechanisms of triplet state riboflavin by tryptophan and tyrosine. *J Photochem Photobiol B*. 2008;92(1):10-12.

3. Martin CB, Tsao ML, Hadad CM, Platz MS. The reaction of triplet flavin with indole. A study of the cascade of reactive intermediates using density functional theory and time resolved infrared spectroscopy. *J Am Chem Soc*. 2002;124(24):7226-7234.

4. Cardoso DR, Libardi SH, Skibsted LH. Riboflavin as a photosensitizer. Effects on human health and food quality. *Food Funct*. 2012;3(5):487-502.
